# A review of neurotoxicities associated with immunotherapy and a framework for evaluation

**DOI:** 10.1093/noajnl/vdab107

**Published:** 2021-11-27

**Authors:** Leeann B Burton, Mahsa Eskian, Amanda C Guidon, Kerry L Reynolds

**Affiliations:** 1 Division of Neuromuscular Medicine, Department of Neurology, Massachusetts General Hospital, Harvard Medical School, Boston, Massachusetts, USA; 2 Division of Oncology, Department of Medicine, Massachusetts General Hospital, Harvard Medical School, Boston, Massachusetts, USA

**Keywords:** CAR-T cells, checkpoint inhibitors, immune effector cell-associated neurotoxicity syndrome (ICANS), immune-related adverse events (irAEs), neurotoxicity

## Abstract

Immuno-oncology agents, including immune checkpoint inhibitors (ICIs) and chimeric antigen receptor T (CAR-T) cell therapies, are increasing in use for a growing list of oncologic indications. While harnessing the immune system against cancer cells has a potent anti-tumor effect, it can also cause widespread autoimmune toxicities that limit therapeutic potential. Neurologic toxicities have unique presentations and can progress rapidly, necessitating prompt recognition. In this article, we review the spectrum of central and peripheral neurologic immune-related adverse events (irAEs) associated with ICI therapies, emphasizing a diagnostic framework that includes consideration of the therapy regimen, timing of symptom onset, presence of non-neurologic irAEs, pre-existing neurologic disease, and syndrome specific features. In addition, we review the immune effector cell-associated neurotoxicity syndrome (ICANS) associated with CAR-T cell therapy and address diagnostic challenges specific to patients with brain metastases. As immunotherapy use grows, so too will the number of patients affected by neurotoxicity. There is an urgent need to understand pathogenic mechanisms, predictors, and optimal treatments of these toxicities, so that we can manage them without sacrificing anti-tumor efficacy.

Immunotherapies have revolutionized the field of cancer therapeutics: the emergence of immune checkpoint inhibitors (ICIs) for solid tumors and chimeric antigen receptor T (CAR-T) cells for liquid tumors has led to tumor response and improved survival in notoriously aggressive cancers.^1–4^ This exciting work has led to Food and Drug Administration (FDA) approval of 8 immune checkpoint inhibitors and 5 CAR-T therapies with numerous other agents under active investigation.^5^ Though the blood-brain barrier has traditionally limited intracranial delivery of chemotherapy, immunotherapies are being increasingly used in patients with brain metastasis.^6^

However, autoimmune toxicities can limit the potent anti-tumor effects of immunotherapies. ICIs disrupt immune tolerance to self-antigens, which has the potential to cause widespread toxicities that lead to permanent discontinuation of therapy and cause significant morbidity and even mortality.^7^ These toxicities, termed immune-related adverse events (irAEs), can involve nearly every organ system including the nervous system. Neurologic irAEs (irAE-Ns) can affect the entire neuroaxis, though have a predilection for the peripheral nervous system.^8^ CAR-T cells can produce both a systemic cytokine release syndrome and neurologic toxicity that can cause encephalopathy, seizures, cerebral edema, and death.^9^ As we treat more patients with immunotherapy and concurrently observe an increase in patients experiencing neurotoxicity, there is an urgent need to further understand the spectrum of irAEs that occur and how to optimally diagnose and manage these patients.

Diagnosing neurotoxicity can be particularly challenging in patients with brain metastasis. There is overlap among presentations of irAE-Ns, tumor progression, and tumor pseudoprogression. Additional challenges relate to the diagnosis of neurologic disease in patients with advanced cancer; for example, differentiating neurologic weakness from generalized fatigue, or evaluating the broad differential for altered mental status in medically sick patients. In this paper, we review neurotoxicity syndromes associated with immunotherapies, with an emphasis on a framework that can be used to distinguish them from other comorbid symptoms in patients with brain metastasis.

## ICI Neurotoxicities

ICIs are monoclonal antibodies against cytotoxic T-lymphocyte-associated protein 4 (CTLA-4; ipilimumab) and programmed cell death protein 1 (PD-1; nivolumab, pembrolizumab, cemiplimab, dostarlimab) or its ligand (PD-L1; atezolizumab, durvalumab, avelumab), given as monotherapy or in combination for a growing list of oncologic indications.^5^ IrAE-Ns are uncommon but clinically important due to the potential for rapid progression, morbidity, and mortality. Early recognition and intervention are crucial in severe cases. Several factors can inform the likelihood that a new neurologic symptom in an ICI-treated patient is an irAE-N, including the therapy regimen, timing of symptom onset, and presence of non-neurologic irAEs or a preexisting neurologic immune-mediated condition.^10^ These general considerations are discussed first, followed by a review of irAE-N syndromes.

### General Considerations

Different ICI regimens confer different risks of irAE-Ns, with dual-ICI combination therapy having the highest risk. A meta-analysis of 59 clinical trials reported irAE-Ns in 3.8% of patients on anti-CTLA-4 inhibitors, 6.1% of patients on anti-PD1 inhibitors, and 12.0% of patients on both in combination.^11^ Most irAE-Ns are mild or moderate, with severe presentations (defined as Common Terminology Criteria for Adverse Events (CTCAE) grade 3 or higher) occurring in <1% of patients.^11^

In patients with brain metastasis, ICIs have been used in combination with stereotactic radiosurgery (SRS) or whole brain radiation therapy (WBRT) to maximize anti-tumor effect.^12^ Data from one prospective and multiple retrospective studies support the safety of radiation and ICIs used either concurrently or sequentially, with low rates of CTCAE grade 4–5 neurotoxicity that were comparable to radiation alone.^12^ However, limitations of prior studies include lack of comparison between single agent ICI and combination radiation/ICI, inability to distinguish radiation-induced neurotoxicity versus ICI, and low numbers of patients on dual-ICI regimens.^12^ Therefore, though safe, whether SRS/WBRT in combination with ICI increases the risk of irAE-Ns is unknown.

Timing and the presence of non-neurologic irAEs are important considerations in evaluating patients for irAE-Ns. Most irAE-Ns occur within 3 months of starting an ICI, though delayed onset has been reported.^13,14^ Suspicion that a new neurologic symptom represents an irAE should be higher when another irAE is present, as multiorgan system involvement is common.^15,16^ In patients with ICI-related neuropathy, 58% had irAEs affecting other organ systems.^17^ Similarly, in a cohort of patients hospitalized with severe irAEs, 21.6% had multiple concurrent toxicities.^16^ Multiple toxicities were most frequent in patients on combination CTLA-4/PD-1 therapy (35.9%), followed by CTLA-4 monotherapy (22.6%) and PD-1/PD-L1 monotherapy (17.2%).^16^

Pre-existing immune-mediated neurologic disease can worsen after ICI treatment. Garcia and colleagues reported 8 cases of patients with preexisting multiple sclerosis who relapsed after ICI treatment; one case was fatal.^18^ ICIs can also cause fatal exacerbation in patients with preexisting myasthenia gravis.^19^ However, the incidence and severity of irAEs have not been studied systematically or prospectively in patients with preexisting neurologic disease, and such patients were excluded from clinical trials. As a result, there may be a bias towards reporting severe exacerbation of preexisting neurologic disorders in the literature. Non-immune-mediated hereditary neuromuscular disorders have been described in ICI-treated patients and can complicate the irAE-N diagnostic work-up; however, there is not a clear link between preexisting non-immune-mediated neurologic disease and increased irAE-N risk.^20^

Whether pre-existing immune-mediated neurologic disease increases the risk of all irAEs is not known. However, patients with other underlying autoimmune diseases (such psoriasis, rheumatoid arthritis, and inflammatory bowel disease) do have higher rates of irAEs. In one cohort of 112 patients, 55% of patients on ipilimumab (CTLA-4) and 38% of patients anti-PD-1/anti-PD-L1 inhibitors had severe (CTCAE grade 3–4) irAEs distinct from symptoms attributable to their pre-existing disease.^21^ For comparison, in all ICI-treated patients, CTCAE grade 3 or higher irAEs occurred in 14% of patients on anti-PD-1 therapy and 34% of patients on anti-CTLA-4 therapy.^22^ Importantly, irAEs in these patients with underlying autoimmunity can typically be managed successfully without requiring treatment discontinuation.^23^ Much is still unknown about how pre-existing neurologic disease affects irAE risk; patients with pre-existing neurologic disease should be monitored closely for symptom exacerbation or de novo irAEs following ICI treatment.

**Figure 1.**
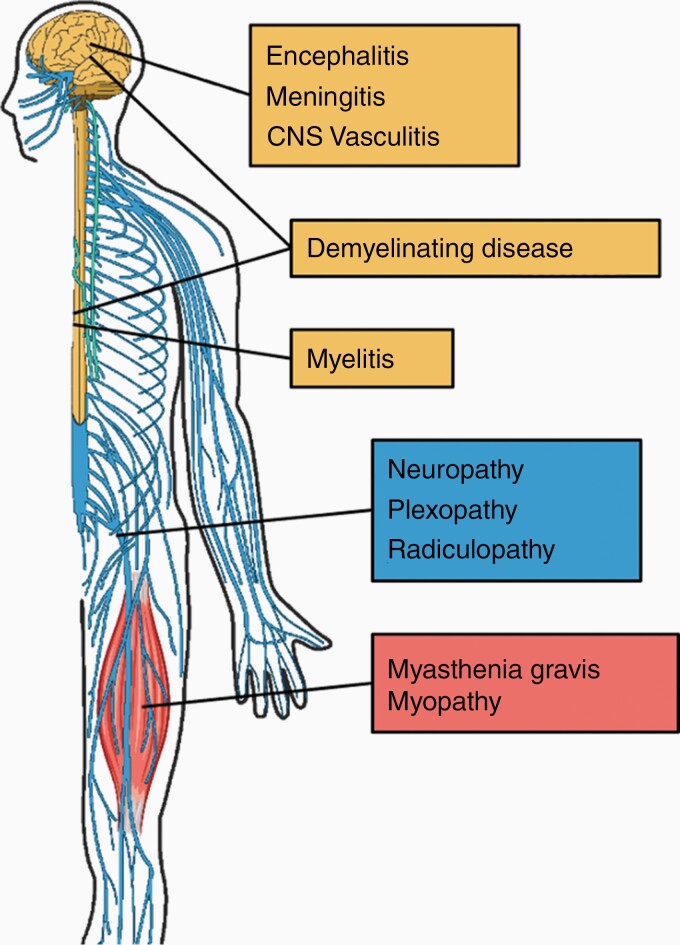
Spectrum of reported neurologic irAEs after ICI therapy.

In the following sections, we describe reported central and peripheral nervous system (CNS, PNS) irAE syndromes, summarized in Figure 1. Though peripheral irAEs are more common, we have ordered the discussion anatomically, starting with the CNS. These classifications are not mutually exclusive, and patients can present with multiple irAE-Ns that can have both CNS and PNS involvement. Notably, the existing literature is limited to retrospective case reports and series. The lack of prospective studies is an important limitation, which makes it difficult to estimate the true incidence and may lead to bias towards severe presentations.

### Central Nervous System irAEs

Identification of CNS irAEs is challenging because many symptoms such as headache, confusion, and fatigue are non-specific and difficult to classify into a neurologic syndrome. As a result, these syndromes may be underrecognized and underreported. Additionally, headache is a presenting symptom of hypophysitis, which is categorized as an endocrine rather than neurologic irAE.^24^ Main categories of reported CNS irAE syndromes include encephalitis, myelitis, demyelinating disease (including optic neuritis), meningitis, and vasculitis.^10^

#### Encephalitis

Immune-related encephalitis (irEncephalitis) is the most reported irAE-N affecting the CNS, with 56 cases in a recent systematic review.^25^ Patients may present with mental status changes, cognitive impairment, seizures, movement disorders, and psychiatric disturbances (ordered highest to lowest frequency).^25^ Recommended work-up includes contrast-enhanced MRI of the brain, lumbar puncture (LP), electroencephalogram (EEG) to assess for subclinical seizures, and autoantibody evaluation.^24^ Diagnosis requires ruling out infectious causes of encephalitis and cytology to rule out disease progression. MRI and LP may show suggestive abnormalities, but can also be unremarkable, making definitive diagnosis challenging in atypical cases. MRI findings include hyperintensity on T2 and fluid attenuated inversion recovery (FLAIR) sequences in the mesial temporal lobes, basal ganglia, and/or cortico-subcortical areas.^25^ Cerebral spinal fluid (CSF) studies are abnormal in most patients, with elevated protein and/or pleocytosis (white blood cells (WBC) usually in the 5–20 cell/mm^3^ range).^25,26^ Patients may have neural-specific autoantibodies, most commonly against Ma-2 and Hu, which can help solidify the diagnosis of immune-mediated encephalitis when the symptoms match antibody-specific syndromes.^25^ These autoantibodies are seen in paraneoplastic syndromes when an immune response is mounted against tumor antigens. Antibodies may be known before ICI treatment, or be detected as part of the irAE-N work-up. It is unclear whether paraneoplastic encephalitis is distinct from irEncephalitis; it has been hypothesized that ICIs unmask subclinical disease by disrupting self-tolerance.^27^

#### Myelitis

Myelitis with or without brain involvement can occur. Motor weakness with paraparesis and gait difficulty is present in nearly all reported cases; other common symptoms included sensory deficits, sphincter dysfunction, and proprioception loss.^25,28^ Recommended work-up includes MRI of the brain and spine, LP, and autoantibody evaluation.^25,28^ Diagnostic test findings mirror those seen in non-ICI-related myelitis. MRI commonly reveals T2 hyperintense lesions, which may be longitudinally extensive spanning 3 or more levels.^25,28^ Patients with longitudinally extensive lesions often have concomitant cord edema and patchy contrast enhancement.^28^ CSF shows an inflammatory pattern, with elevated protein (median 80, range 50–310 mg/dL in a 7-case series) and lymphocytic pleocytosis (median WBC count 24, range 3–102 cells/uL) in most patients and oligoclonal bands (OCBs) in a subset.^25,28^ Autoantibodies are typically negative, though antibodies against glial fibrillary acidic protein (GFAP) and collapsin response-mediator protein-5 (CRMP-5) have been reported.^28,29^ Antibodies against aquaporin-4 (AQP4) or myelin oligodendrocyte glycoprotein (MOG) are typically negative, though when present these patients are categorized as having demyelinating disease rather than myelitis.^25,28^ Interestingly, a substantial number of patients who develop myelitis have a history of thoracic radiation exposure (43% in one 7-patient series, and 23% in a systematic review), though it is unclear if radiotherapy is a true predisposing factor.^28^

#### Demyelinating disease

Demyelinating disease in the CNS includes multiple sclerosis, neuromyelitis optica spectrum disorder (NMSOD), transverse myelitis, and isolated optic neuritis, all of which have been reported after ICI treatment.^29^ Symptoms can occur due to pre-existing disease exacerbation or de novo disease and vary based on the region affected, including weakness, sensory symptoms, vision loss, and cognitive dysfunction.^25,29^ Optic neuritis is rare, but when present can be painless (in contrast the typical non-ICI-related presentation) and affects both eyes in 64% of patients.^30^ Demyelinating disease can manifest with brain and/or spinal cord lesions, so clinical features overlap with encephalitis and myelitis, without consistent methods for differentiating them in published case series. As in encephalitis and myelitis, the work-up includes MRI of the brain and spine, LP, and autoantibody evaluation. In cases of suspected optic neuritis, MRI of the orbits may also be performed. In general, MRI lesions are considered demyelinating based on the presence of T2 hyperintensity and/or contrast enhancement and location in cortical, juxtacortical, periventricular, or infratentorial regions intracranially or in the spinal cord (McDonald criteria for multiple sclerosis^31^). MRI of the orbits can show optic nerve enhancement, typically sparing the retrobulbar and proximal segment.^29,30^ CSF is usually abnormal: in an 18-case series, 78% had elevated protein (median 93.5, range 50–380 mg/dL), 56% had pleocytosis (median WBC count 22, range 14–1195 cells/mm3), and 39% had OBCs.^29^ AQP4 antibodies may be positive, particularly in patients presenting with a longitudinally extensive transverse myelitis.^25^ Though idiopathic demyelinating disease is often relapsing and remitting, ICI-related disease typically has a monophasic course and does not relapse after ICI discontinuation and treatment for initial symptoms.^25^

#### Meningitis

Aseptic meningitis can mimic infectious meningitis, presenting with headache, neck stiffness, fever, nausea/vomiting, photophobia, and symptoms of increased intracranial pressure (such as visual obscurations, diplopia, or pulsatile tinnitus).^25,32^ Patients with meningitis should have preserved level of consciousness and cognitive function.^30,32^ The presence of altered mental status in many reported cases may suggest parenchymal involvement (i.e., encephalitis rather than meningitis), which is a limitation in the literature.^25^ Patients require lumbar puncture to rule out an infectious meningitis. MRI brain is also recommended, which can show leptomeningeal enhancement but is also important for ruling out other etiologies (e.g., vascular events, metastasis, hypophysitis).^24,32^ Supportive CSF findings include pleocytosis (median WBC count 143, range 20–705 cells/uL in a 13-case series), elevated protein (median 150, range 60–500 mg/dL), normal glucose, and negative infectious studies.^25,32^ MRI may reveal leptomeningeal enhancement (31% in a 13-case series), leptomeningeal T2 hyperintensity (15%), or nerve root enhancement (8%).^25^ In patients with brain metastasis, leptomeningeal disease is an important alternative diagnosis and can be assessed with CSF cytology/flow cytometry or biopsy in some cases. Of note, serial LPs with repeated cytology are performed initial testing is negative to increase sensitivity: the first LP is 50–60% sensitive for malignant cells; a second LP increases sensitivity to 80%; additional LPs increase sensitivity by 2–5% per collection.^33^

#### Vasculitis

There are several case reports of vasculitis after ICI treatment, including primary angiitis of the central nervous system (PACNS).^34^ PACNS is a rare type of vasculitis that is isolated to the brain and spinal cord and can present with headache, stroke, seizure, encephalopathy, and focal neurologic deficits depending on the region involved. Diagnosis requires demonstration of abnormal vessels with angiography (conventional or MRI/CT-based), vessel wall imaging, or biopsy. Work-up also includes MRI brain to assess for infarction, LP, and serum markers associated with systemic vasculitis (including ANCA, ANA, ESR, CRP, cryoglobulins). Vessel abnormalities include narrowing or beading on angiography, concentric vessel wall enhancement on vessel wall imaging, and/or granulomatous, lymphocytic, or necrotizing vasculitis on biopsy.^30,35^ There is limited published data to determine expected findings in ICI-treated patients, but studies in non-ICI-related PACNS are informative. Most patients have abnormal CSF with mild elevations in WBC count and protein, as well as non-specific MRI abnormalities that include cortical and subcortical infarction, parenchymal and leptomeningeal enhancement, and T2/FLAIR hyperintensity.^35^ Notably, patients with brain metastasis who undergo radiation can develop radiation vasculopathy; whether brain radiation increases the risk of ICI-related vasculitis is unknown.

In addition to the syndromes described above, case reports exist of posterior reversible encephalopathy syndrome (PRES) occurring in the context of ICI treatment.^35,36^ PRES, which may present with headache, seizures, altered mental status, and vision loss, is thought to occur due to endothelial dysfunction in the context of medications, systemic hypertension, and eclampsia. The connection between PRES and immune dysregulation with ICI use is unclear. Although debate exists about whether PRES is an irAE-N, clinicians should be aware of PRES as a potential occurrence in the context of ICI treatment.^30^

### Framework for Diagnosing IrEncephalitis in Patients with Brain Metastases

**Figure 2.**
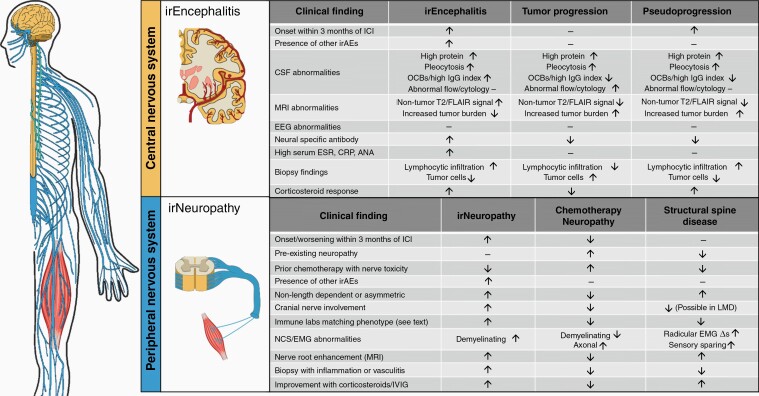
Clinical findings that increase the likelihood of (1) irEncephalitis versus tumor progression and pseudoprogression (top) and (2) irNeuropathy versus chemotherapy-induced neuropathy (bottom).

In patients with brain metastasis, differentiating irEncephalitis from alternative diagnoses of tumor progression and pseudoprogression is challenging. Blood-brain barrier breakdown can occur in all three, leading to similar diagnostic test findings. Pseudoprogression, defined as a transient increase in tumor burden with subsequent tumor shrinkage, occurs after brain radiation and has also been described after ICI treatment in 10–20% of patients.^37^ Characteristics that make each of these diagnoses more or less likely are summarized in Figure 2.

Onset of symptoms within 3 months of ICI initiation supports irEncephalitis. Pseudoprogression occurs in the same post-treatment time window, remaining a diagnostic consideration when radiographic changes emerge within 3 months of ICI treatment. However, irEncephalitis and pseudoprogression are distinguishable based on clinical grounds. While irEncephalitis presents with clinical deterioration in combination with radiographic change, the radiographic abnormalities seen in pseudoprogression are more likely to be asymptomatic: retrospective studies in glioma patients have noted symptoms in 21–33% of patients with pseudoprogression.^38–41^ This is straightforward in theory but can be murky in practice; medically complex patients often have multiple potential etiologies for acute or sub-acute cognitive changes. Clinical assessment and neuroanatomical correlation are critical to determine whether neurologic symptoms can be attributed to a changing radiographic lesion.

The presence of non-neurologic irAEs increases the likelihood of irEncephalitis given the high rate of multisystem involvement. However, non-neurologic irAEs are common so their presence does not definitively rule out tumor progression or pseudoprogression.

CSF analysis is helpful in diagnosing irEncephalitis, but abnormalities overlap with those seen in tumor progression and pseudoprogression. Though CSF findings of elevated protein and pleocytosis are common in irEncephalitis, these findings are not specific and occur in patients with brain metastases due to breakdown of the blood-brain barrier. The presence of CSF OCBs and/or an elevated IgG index indicates increased intrathecal immunoglobulin synthesis and suggests irEncephalitis, though this is not sensitive (present in 24% of irEncephalitis cases).^25^ Of note, OCBs have been reported in brain tumors, but are uncommon: in a cohort of 270 patients with positive OCBs, only 5 patients (1.8%) had a brain tumor.^42^ Finally, the presence of malignant cells on cytology and/or flow cytometry make tumor progression more likely, though this finding can be seen in irEncephalitis or tumor pseudoprogression if leptomeningeal metastases are present.

On MRI, the presence of T2/FLAIR hyperintensity in areas without active tumor (i.e., beyond the area of peritumoral vasogenic edema) is a key feature that differentiates irEncephalitis. Though more sophisticated imaging techniques have been used to distinguish tumor progression and pseudoprogression, we have not included them due to limited data on their use irEncephalitis. EEG may reveal seizure activity, epileptiform discharges, or slowing, though any of these non-specific findings may be seen in all 3 conditions.

The presence of a neural-specific autoantibody that aligns with the clinical syndrome also increases the likelihood of irEncephalitis, though non-specific low titer positivity can be seen in patients treated with ICIs. The higher the antibody titer, the more convincing it is that irEncephalitis is the cause of the presenting symptoms.^10^ Elevations in ESR, CRP, and ANA titer are non-specific markers of inflammation of unclear clinical significance in isolation, though, when abnormal, may suggest an autoimmune tendency and support a diagnosis of irEncephalitis when considered in conjunction with other data.^10,24^

Limited reports of brain pathology in patients with irEncephalitis show perivascular CD8+ lymphocytic infiltrate.^43,44^ Notably, histopathological findings of pseudoprogression in the brain related to ICI treatment are similar.^37^ While biopsy cannot distinguish between irEncephalitis and pseudoprogression, the presence of lymphocytic infiltrate in the absence of tumor cells makes tumor progression unlikely. Finally, response to corticosteroids increases the likelihood of both irEncephalitis and pseudoprogression.

In summary, though findings in irEncephalitis, tumor progression, and pseudoprogression often overlap, features that suggest a diagnosis of irEncephalitis include: onset of new or worsening neurologic symptoms within 3 months of starting an ICI, the presence of concurrent non-neurologic irAEs, CSF OCBs/high IgG index, T2/FLAIR hyperintensity on MRI in regions without tumor, the presence of neural specific autoantibodies, and elevated serologic inflammatory markers. Identification of biomarkers and diagnostics for irAE-Ns is ongoing and essential to improve the accuracy of the diagnosis and determine best treatments.

### Peripheral Nervous System irAEs

Neurologic irAEs have a predilection for the PNS, which is affected twice as often as the CNS.^26^ Symptoms can manifest as neuropathy, myasthenia gravis, and/or myopathy. These irAEs often behave differently than non-ICI variants and can have fulminant presentations that are important for clinicians to recognize.^8^

#### Neuropathy

Immune-related neuropathy (irNeuropathy) has a highly variable phenotype, including (in order of frequency) cranial neuropathies with or without meningitis, non-length-dependent polyradiculoneuropathy, and acute sensorimotor axonal neuropathies.^17,25,45^ Less commonly reported irNeuropathy phenotypes include sensory neuronopathy, neuralgic amyotrophy, small fiber/autonomic neuropathy, vasculitic neuropathy, plexopathy/radiculoplexopathy, and mononeuritis multiplex.^17,25^ Work-up may include serologic testing to evaluate for reversible neuropathy causes, nerve conduction studies (NCS) with electromyography (EMG), lumbar puncture, and MRI of the spine (to assess for nerve root enhancement/thickening and rule out compressive etiology); however, all of these modalities may not be required, and work-up is tailored to the patient and phenotype.^24^ To date, no relationship has been found between irNeuropathy phenotype and history of preexisting neuropathy or neurotoxic chemotherapy exposure.^45^ Because clinical features and diagnostic findings are quite different across irNeuropathy phenotypes, the most common types are discussed individually.

Cranial neuropathy can occur in isolation or in conjunction with concurrent meningitis, encephalitis, or demyelinating polyneuropathy (such as Guillain Barre syndrome, GBS).^17,46^ Importantly, the differential also includes leptomeningeal carcinomatosis, which needs to be excluded. Facial nerve involvement is most frequently reported (either unilaterally or bilaterally), though involvement of the oculomotor, trigeminal, abducens, vestibulocochlear, and glossopharyngeal nerves can occur, and multiple cranial nerves may be involved.^17,25,46^ (In this review we have considered the optic nerve part of the CNS; optic neuritis is discussed as part of Demyelinating Disease). MRI commonly shows cranial nerve enhancement, and CSF analysis may show elevated protein, leukocytosis, and OCBs.^17,25^

Demyelinating polyradiculoneuropathy can present acutely (i.e., GBS), sub-acutely, or chronically (i.e., chronic inflammatory demyelinating polyneuropathy, CIDP).^17^ Patients may present with non-length-dependent sensory symptoms (e.g., not progressing in a distal to proximal pattern; possibly involving the face, upper extremities, torso, and proximal lower extremities), weakness, back pain, concomitant cranial neuropathies, respiratory compromise, and dysautonomia.^17,25^ The GBS variant of Miller Fisher syndrome, characterized by ophthalmoplegia, ataxia, and areflexia, has also been reported.^46^ By definition, nerve conduction studies (NCS) in demyelinating neuropathies illustrate these demyelinating features, though may also show secondary axonal loss. The most common abnormalities include prolonged distal motor latencies and slowed conduction velocities, and lower extremities tend to be more affected than upper extremities.^45^ Many patients (49% of patients with irNeuropathy in a systematic review) show CSF cytoalbuminologic dissociation typical of GBS and CIDP, though elevations in both protein and WBC was more common than in non-ICI GBS, present in 34% (median WBC of 11 cells/uL).^25^ Nerve root enhancement on MRI was common.^17,25^ Notably, patients presenting with GBS have been treated successfully with both IV steroids and IVIG, whereas IV steroids are typically avoided in non-ICI GBS.

IrNeuropathy can be axonal rather than demyelinating. Axonal sensory or sensorimotor polyneuropathy often presents with non-length-dependent symptoms, in contrast to the length-dependent or distal symmetric pattern characteristically seen in non-ICI axonal polyneuropathy.^17^ NCS/EMG show axonal loss without demyelinating features. CSF studies are not consistently reported for this subgroup. Patients with axonal irNeuropathy often have significant neuropathic pain, which differs from the demyelinating phenotype.^17,45^ Of note, infliximab exposure can rarely cause a painful non-length-dependent small fiber neuropathy that presents similarly.^47^ Infliximab is frequently used in the treatment of non-neurologic steroid-refractory irAEs, and should therefore be considered in the differential if applicable. Chemotherapy-related neuropathy, either given before or concurrently with ICI, also has an axonal phenotype and should be considered in the differential (discussion to follow).

#### Neuromuscular junction disorders

Myasthenia gravis (MG) can occur after ICI treatment, either due to preexisting disease exacerbation or de novo disease. In a series of 65 patients with ICI-related MG, 20% had pre-existing MG while 80% presented with de novo disease.^48^ Common presenting symptoms include ptosis, diplopia, dysphagia, dyspnea, and limb weakness.^48^ ICI-related MG tends to be severe, with high reported rates of bulbar involvement (75%), respiratory failure (45–65%), and death (38%).^25,48^ Patients can present with concurrent myopathy and myocarditis, with overlap being associated with severe disease.^25,49^ In a 12-patient case series of nivolumab-related MG, 1/3 had muscle involvement.^49^ Recommended work-up includes myasthenia antibody evaluation (including antibodies against acetylcholine receptor (AChR) and muscle-specific tyrosine kinase (MuSK)), creatine kinase (CK) to screen for concomitant myopathy, NCS/EMG (including repetitive nerve stimulation to assess for MG and needle exam to assess for myopathy), and pulmonary function testing to evaluate for respiratory involvement.^24^ Positive AChR antibodies are present in 59–66% of patients with generalized weakness, and more likely to be positive in patients with pre-existing MG.^25,48^ Antibodies against P/Q type voltage-gated potassium channels, seen in Lambert Eaton myasthenic syndrome, have also been reported and may be paraneoplastic in etiology.^25^ Electrodiagnostic studies demonstrating decrement on repetitive nerve stimulation or increased jitter on single fiber EMG support the diagnosis.^49^ Needle EMG may additionally show abnormal spontaneous activity and/or short duration, sometimes polyphasic motor unit potentials in patients with overlapping myopathy.

#### Myopathy

Immune-related Myopathy (irMyopathy) is the most frequently described irAE-N. This may be called irMyositis or necrotizing myopathy in the literature. Patients present with acute or subacute myalgias and limb-girdle, axial, bulbar, and/or ocular motor weakness.^50,51^ Notably, weakness in ocular, facial, and respiratory muscles, cardiac involvement (myocarditis), and myalgias were more frequent in irMyopathy compared to controls with necrotizing myopathy associated with signal recognition particle (SRP) antibodies or anti-synthetase syndrome.^51^ irMyopathy can also be focal and asymmetric, which is not typical for most other inflammatory myopathies.^50^ The clinical presentation can mimic MG by involving ocular, bulbar, neck, and respiratory muscles, so it can be difficult to distinguish ICI-related myopathy and MG, particularly when overlap exists. Recommended work-up includes serum testing for evidence of muscle inflammation and myositis-specific antibodies, screening for concurrent myocarditis, EMG, and in some cases muscle biopsy.^24^ Serum creatine kinase (CK) is usually elevated (5247 IU/L on average in one cohort of 19 patients) but maybe normal and in our experience is not a reliable biomarker for disease severity or improvement.^50–52^ Myositis-specific antibodies are usually negative, though anti-striational antibodies are often positive.^25,51^ EMG and MRI can add helpful information: in a systematic review of 136 reported cases, MRI showed muscle edema and other findings of myositis in 85% and myopathic EMG findings were present in 49%.^25^ In our experience, EMG abnormalities can be isolated to thoracic paraspinals, making these muscles important to include in electrodiagnostic evaluation. The paraspinal muscles are also frequently involved in MRI.^53^ When performed, muscle biopsy typically shows necrosis with endomysial inflammation consisting of CD8+ T-cells.^25,50,51^

The overlap between ICI-related MG, myopathy, and myocarditis is important to recognize because patients’ strength and respiratory function can decline rapidly and mortality rates are high. In patients with myopathy, concurrent myocarditis is present in 16.1–40% depending on the cohort.^50–52,54,55^ Rates of myocarditis are similar in patients with ICI-related MG, and these patients are more likely to have severe disease and require respiratory support.^25,49^ ICI-related myocarditis is often severe, with 46% of patients in one multicenter cohort experiencing a major adverse cardiac event (defined as cardiovascular death, cardiogenic shock, cardiac arrest, or hemodynamically significant heart block).^15^ The fatality rate for ICI-related myocarditis has been estimated at 27–40%, which is substantially higher than the rate of 4% reported for patients with non-ICI myocarditis.^54^ Patients with overlap syndromes require aggressive treatment and benefit from multidisciplinary care.

### Framework for Diagnosing irNeuropathy in Patients with Chemotherapy Exposure

Diagnosis of irNeuropathy may be complicated by other factors in patients with metastatic cancer, including prior or concomitant exposure to neurotoxic chemotherapy. Common neurotoxic chemotherapies include taxanes and platinum-based agents. In patients on concomitant ICI and chemotherapies, identifying the culprit medication is particularly critical if symptoms are severe enough to warrant discontinuation. There are clinical features that distinguish irNeuropathy from chemotherapy-related neuropathy, summarized in Figure 2.

As in other neurologic irAEs, the acute or subacute onset of neuropathy symptoms within 3 months of starting an ICI and the presence of non-neurologic irAEs are features suggestive of irNeuropathy. Neuropathy associated with chemotherapy has a more chronic course and maybe present before ICI initiation.^17^ Neuropathic pain is often seen in chemotherapy-related neuropathy. Though not characteristic of demyelinating irNeuropathy phenotypes, neuropathic pain is a prominent symptom in axonal irNeuropathy.^17,45^

The pattern of involvement differs between irNeuropathy. Chemotherapy-related neuropathy is more likely to be length-dependent (starting in the distal lower extremities and progressing proximally) and unlikely to be associated with cranial nerve involvement.^17^ Though length-dependence does not rule out an irNeuropathy, non-length-dependent or highly asymmetric presentations essentially rule out a chemotherapy-related neuropathy. Both types, however, can present with a non-length dependent sensory neuronopathy phenotype. Similarly, while axonal pathology on NCS does not rule out irNeuropathy, demyelinating features are suggestive. Serum and CSF diagnostic studies can also support irNeuropathy when abnormalities correspond to the neuropathy phenotype: for example, positive ANCA in mononeuritis multiplex or CSF cytoalbuminologic dissociation in GBS. MRI may show nerve root enhancement and biopsy may show lymphocytic inflammation or vasculitis in irNeuropathy, which would not be expected in chemotherapy-related neuropathy.

### Severity Grading, Management, and Outcomes

CTCAE is used to grade the severity of irAE-Ns, though these criteria may not adequately capture irAE-N severity due to the potential for rapid progression and very focal but severe disease.^56^ Grading is as follows: grade 1 is asymptomatic or mild disease; grade 2 is moderate disease that limits age-appropriate instrumental activities of daily living (IADLS); grade 3 is severe disease that limits self-care and may require hospitalization; grade 4 is life-threatening disease requiring urgent intervention; grade 5 is death.^57^ CTCAE grade does not always accurately reflect irAE-N severity. New severity criteria specifically designed to grade irAE-Ns were recently published.^10^

IrAE-Ns span the entire range of severity and can be fatal.^7,25^ Reported mortality rates by syndrome from a systematic retrospective case review were as follows: myasthenic syndromes 28%, encephalitis 21%, myositis 17%, CNS demyelinating diseases 12%, GBS 11%; mortality was 0% for cranial neuropathies, meningitis, and myelitis.^25^ Key warning signs that may signal severe disease in the CNS include rapidly progressive altered mental status, decreased level of consciousness, refractory seizure activity (including status epilepticus), and signs of increased intracranial pressure (headache, blurred or double vision, vomiting). In the PNS, respiratory compromise is a life-threatening complication of neuropathy, myasthenia, and myopathy, so complaints of dyspnea, dysphagia, and signs of orthopnea or increased work of breathing should be taken seriously. Bulbar and neck flexor weakness can also signal more severe presentations. Finally, myocarditis should be considered in patients presenting with weakness due to either myasthenia or myopathy. Presentation with any of the above symptoms prompts urgent or emergent inpatient work-up and management, as 46% of patients with ICI-associated myocarditis go on to develop major adverse cardiac events including cardiogenic shock, cardiac arrest, complete heart block, or death.^15^

There are multiple consensus guidelines for management of irAE-Ns, including from ASCO in 2018 and NCCN in 2021.^24,58^ Treatment is largely determined by severity grading. Though there are disease-specific considerations, in general, mild disease is monitored clinically, ICI is held in moderate disease and corticosteroid treatment is often initiated, and hospitalization and IV glucocorticoid treatment is considered in grades 3 and higher.^24^ Alternative immunomodulating and/or immunosuppressive therapies including intravenous immunoglobulin (IVIG), therapeutic plasma exchange (TPE), and rituximab may be considered as appropriate for the syndrome.^59^ Most patients do improve with discontinuation of ICI and treatment with corticosteroids or other therapies. Of 35 irAE-N cases reported from a pharmacovigilance database, 75% had documented resolution.^60^ However, persistent deficits are possible, particularly if there is structural injury (i.e., strokes in vasculitis or axonal loss in neuropathy).

The relationship between irAEs and ICI anti-tumor efficacy has not been fully elucidated. IrAEs may represent collateral damage from activated T-cells, which is also the mechanism of the anti-tumor response. Therefore, the presence of irAEs may be a marker of anti-tumor activity. Indeed, there is a growing body of evidence linking irAEs to marked improvements in progression-free survival, overall survival, and overall response rate, but these findings are not consistently replicated.^61^ This is an area of active research. Understanding the link between toxicity and efficacy is crucial as we seek to mitigate toxicities while optimizing the anti-tumor effects of these potentially lifesaving medications.

## CAR-T Cell Neurotoxicity

CAR-T cell therapy uses patient-derived T-cells, modifies the cells in a lab to express a chimeric antigen receptor (CAR) to target the tumor, and reinfuses the cells into the patient after conditioning chemotherapy to elicit an anti-tumor response. The CAR includes an antigen binding domain that binds tumoral antigens, ultimately leading to tumor cell lysis. Four currently approved CAR-T cell therapies target CD19, which is a surface molecule primarily expressed on B-cells: tisagenlecleucel, axicabtagene ciloleucel, brexucabtagene autoleucel, and lisocabtagene maraleucel. In addition, the most recent CAR-T cell therapy approved, idecabtagene vicleuce, targets B-cell maturation antigen (BCMA; expressed on plasma cells) in patients with multiple myeloma. Indications for CAR-T cell therapies are currently limited to hematological malignancies; however, application to solid tumors is a very active area of investigation. As indications expand, recognizing CAR-T related neurotoxicity and differentiating it from brain metastasis will become increasingly important.

### Immune Effector Cell-Associated Neurotoxicity Syndrome

Unlike irAE-Ns related to ICIs, CAR-T neurotoxicity, termed immune effector cell-associated neurotoxicity syndrome (ICANS), is common. The overall incidence of ICANS (any severity grade) is 23–67% in patients with lymphoma and 40–62% in patients with leukemia.^62^ Symptoms include (in order of decreasing frequency, based on a cohort of 33 patients): encephalopathy/delirium, aphasia, depressed level of consciousness, seizure, headache, tremor/myoclonus, motor dysfunction, dysarthria, neuropathy, meningismus, hemiparesis, and hallucinations.^63,64^ Interestingly, aphasia is a characteristic and early symptom seen in severe ICANS.^63^ Diagnosis is based on clinical grounds as delineated in a consensus grading system from the American Society for Transplantation and Cellular Therapy (ASTCT), which defines grades 1–4 (4 being most severe) based on the presence of encephalopathy (measured by the Immune Effector Cell Encephalopathy (ICE) score), depressed level of consciousness, seizures, motor findings, and/or elevated intracranial pressure/cerebral edema.^65,66^ The grading criteria are outlined in Table 1. Additional work-up may include serologic testing for coagulopathy (which can occur in severe ICANS), neuroimaging with CT or MRI, and EEG.^65^ Neuroimaging is unremarkable in most cases, but shows cerebral edema when present; other patterns of FLAIR/T2 hyperintensity in the bilateral thalami and brainstem, multifocal microhemorrhage, leptomeningeal enhancement, and transient corpus callosum lesions have also been reported.^63–65^ EEG may reveal non-convulsive seizures or non-specific slowing that can be seen in other causes of encephalopathy.^65^ Serum and CSF cytokine levels and markers of endothelial activation have been proposed as biomarkers for diagnosis and monitoring. These tests, however, are not widely available and their long turn-around time currently limits clinical utility.^67^

**Table 1.** Grading and Treatment of Neurotoxicity in CAR-T Cell Therapy (Modified From American Society for Transplantation and Cellular Therapy and Society for Immunotherapy of Cancer Guidelines^65,66^)

Sign or Symptom	Grade 1	Grade 2	Grade 3	Grade 4
Impaired Consciousness	Awakens spontaneously	Awakens to voice	Awakens only to tactile stimulation	Unarousable or requires vigorous or repetitive tactile stimuli to arouse; stupor or coma
Cognitive Impairment	ICE score 7–9	ICE score 3–6	ICE score 0–2	ICE score 0. Patient is unarousable; cannot perform ICE
Motor weakness	Not present	Not present	Not present	Deep focal motor weakness such as hemiparesis or paraparesis
Seizure	Not present	Not present	Focal or generalized seizure that resolves rapidly; non-convulsive seizures on EEG that resolve with intervention	Life-threatening prolonged seizure (>5 min); repetitive clinical or electrical seizures without return to baseline in between
Elevated Intracranial Pressure/Cerebral Edema	Not present	Not present	Focal/local edema on neuroimaging	Diffuse cerebral edema on neuroimaging; decerebrate or decorticate posturing; cranial nerve VI palsy; papilledema; Cushing’s triad
Treatment	Clinical monitoring^a^	Clinical monitoring; consider corticosteroids^b^	Consider ICU monitoring; corticosteroids; levetiracetam for seizures	ICU monitoring; corticosteroids; levetiracetam for seizures; hyperosmolar therapy for intracranial hypertension

EEG, electroencephalogram; ICE, immune effector cell encephalopathy; ICU, intensive care unit.

^a^Clinical monitoring includes vitals, exam, and encephalopathy screening (ICE) every shift.

^b^Typical corticosteroid dose is dexamethasone 10 mg every 6 h; fast taper should be used once there is improvement.

Cytokine release syndrome (CRS) precedes ICANS in most (but not all) cases, so can be considered an initiating event for ICANS.^68^ CRS, the most common adverse event related to CAR-T cell therapies, is characterized by systemic constitutional symptoms due to elevated serum cytokines and generalized immune activation in response to CAR-T cell activation and expansion.^65,68^ These symptoms include fever and malaise, and in more severe cases hypotension, hypoxia, and multi-organ failure.^65^ CRS usually occurs in the first week after CAR-T cell infusion, with ICANS occurring subsequently in the second week after CRS symptoms have subsided, though CRS and ICANS can overlap.^68^ The timing of ICANS onset after CAR-T cell infusion can range from 1 day to 3–4 weeks, with a median of 5–7 days.^62,63,69,70^

In addition to CRS, risk factors for ICANS include high pre-treatment disease burden and lymphoma subtype, extramedullary disease, younger-age, pre-existing neurologic comorbidities, CAR-T cell construct and dose, high peak levels of CAR-T expression, fever, cytopenias, high levels of circulating inflammatory markers (including CRP, ferritin, procalcitonin) and cytokines (though many cannot be measured commercially.^64,65,71^ Work is underway to develop predictive biomarkers for ICANS. In a cohort of 45 patients treated with axicabtagene ciloleucel (25 of whom developed ICANS), pre-infusion fibrinogen level was predictive of ICANS (odds ratio 2.68 for any grade ICANS; odds ratio 3.27 for severe ICANS).^70^ Rubin and colleagues also developed a multivariate prognostic score for determining ICANS probability based on data available within the first 5 days of admission, which when tested on a separate validation cohort performed with 77% accuracy, 82% sensitivity, and 70% specificity.^71^ Tools for predicting which patients are likely to develop ICANS will inform clinical monitoring strategies and may enable a shift from reactive to preventative treatment.

ICANS is primarily treated with corticosteroids, levetiracetam for seizures, supportive care including close monitoring in an intensive care unit, and management of cerebral edema in severe cases.^65^ Though treatment with tocilizumab (a monoclonal antibody that blocks the interleukin-6 (IL-6) receptor) is standard of care in CRS, there is no proven benefit in ICANS and some hypothesize that tocilizumab can worsen neurotoxicity by increasing CSF IL-6 levels.^62,64,65^ As such, use of tocilizumab in ICANS without CRS is not recommended.^65,72^ Though severe ICANS can occur, prognosis is favorable with proper identification and management. Most patients have complete recovery within 2 months.^64,69^ Fatal ICANS, particularly from cerebral edema, can occur.^9,65^ In one French cohort, 13% of patients with neurotoxicity died; however, cause of death was not definitively related to neurotoxicity as some patients had concomitant tumor progression and/or sepsis.^69^ Other reported fatality rates from ICANS range from 0 to 32%.^9,63,64^

Unfortunately, while necessary to treat ICANS, steroids are associated with decreased progression-free and overall survival.^73^ New treatment approached are needed to treat ICANS without compromising the anti-tumor effect. Medications such as anakinra (IL-1R antagonist), lenlizumab (anti-GM-CSF), and siltuximab (anti-IL-6, which have better blood-brain barrier penetration than tocilizumab) are being studied as alternative therapies.^62,74^ ICANS management has been reactive to date; studies are also underway to investigate whether prophylactic treatment with anti-cytokine therapies can prevent neurotoxicity (NCT04150913, NCT04148430).

Though acute CAR-T-related neurotoxicity can be severe, data from survivors suggest limited long-term neurologic sequelae. In a survey sent to long-term survivors of CAR-T cell therapy at 1–5 years post-treatment, 35.7% reported cognitive difficulties (including difficulties with memory, word finding, concentration, and problem solving); however, this may be unrelated to CAR-T cell treatment.^75^ This rate is similar to self-reported rates of cognitive impairment in cancer survivors who did not receive CAR-T cell therapy, and baseline rates were not available in this cohort.^75^ Maillet and colleagues recently published results of baseline and 6–12-month follow-up neurologic and cognitive assessments in a cohort of 27 patients with CAR-T cell-treated B-cell lymphoma without tumor progression at follow-up.^76^ Twelve of these patients had ICANS. In all patients, follow-up neurologic and cognitive assessments at 6–12 months post-treatment were unchanged from baseline.^76^

### ICANS in Patients with Brain Metastases

To date, CAR-T cell therapy has been used cautiously in patients with known CNS involvement of lymphoma or leukemia due to safety concerns. Data on the risk of neurotoxicity in patients with pre-existing CNS disease are mixed. Though these patients were excluded from clinical trials, a cohort of 8 patients with secondary CNS lymphoma treated with tisagenlecleucel did not have higher risk of neurotoxicity.^77^ However, in retrospective cohorts, pre-existing neurologic disease has emerged as a possible risk factor for ICANS. In 133 patients who developed ICANS after CAR-T cell treatment, the presence of any preexisting neurologic comorbidity (present in 58/133) was significantly associated with neurotoxicity (*P* = 0.0059, relative risk not reported).^64^ Preexisting peripheral neuropathy, CNS tumor involvement, seizures, cognitive impairment, intracranial hemorrhage, and headache disorders were included. When analyzed by specific neurologic disease type, no single type was significantly associated; however, low numbers may have resulted in insufficient power.^64^ More data to clarify the relationship between pre-existing neurologic disease and ICANS will be important as CAR-T cell therapy applications expand to include more patients with neurologic disease.

The presence of brain metastasis may also pose additional challenges when diagnosing ICANS. Even in patients without brain disease, clinical evaluation of patients with severe ICANS on corticosteroids can be difficult; for example, the differential for worsening encephalopathy includes refractory ICANS, steroid effects, infection, ICU delirium, other metabolic factors that occur in cytokine release syndrome, or a combination. Patients with a poor neurologic substrate due to brain disease are often more susceptible to metabolic encephalopathy and may have predisposition to seizures before CAR-T cell infusion. This underscores the need for clinically available biomarkers to aid clinical care. Additionally, progression of brain metastases would need to be considered in the differential for this patient group. However, many clinical features can distinguish ICANS from tumor progression, including symptom onset in the days to weeks after CAR-T cell infusion, rapid progression of symptoms, concomitant systemic cytokine release syndrome, MRI with edema in the absence of a mass lesion, and resolution of symptoms with treatment.

### CAR-T Cell Neurotoxicity in Solid Tumors

Though CAR-T cell indications are currently limited to hematologic malignancies, trials have investigated the safety and efficacy in solid tumors including glioblastoma (GBM). A major barrier to application of CAR-T cells to solid tumors has been the identification of tumor-specific antigen targets that are not expressed on normal tissues.^78^

In non-CNS solid tumors, the rates of neurotoxicity appear to be low. No neurotoxicity was observed in small phase 1 trials testing CAR-T cells in breast cancer and prostate cancer.^79,80^ Among the 3 trials evaluating CAR-T cells in colorectal cancer, one trial reported adverse events of headache and paresthesia (in 20% and 30% of patients, respectively), but no patients developed ICANS; in the other 2 trials no neurotoxicity was reported.^81–83^ Clinical efficacy of these constructs remains to be shown.

Neurologic adverse events have been reported in patients treated with experimental CAR-T cells for GBM; however, because neurologic events are common in patients with GBM, there is ambiguity regarding causality. Two phase 1 studies evaluated CAR-T cells targeting epidermal growth factor receptor variant III (EGFRvIII) in patients with GBM.^84,85^ In one trial, 2 of 18 patients developed grade 3 or 4 serious neurologic adverse events (1 with transient motor weakness and 1 with transient urinary incontinence).^84^ Additionally, 10 patients required imaging for worsening grade 2 neurologic symptoms or suspected seizure activity.^84^ In the other trial, 3 of 10 patients developed clinically significant neurologic events, including: seizure with altered mental status that resolved over several days after treatment with anti-epileptics, steroids, siltuximab, and correction of hyponatremia; neurologic decline that was most consistent with progressive disease; neurologic decline post-operatively after tumor resection that was attributed to hemorrhage in the surgical bed.^85^ Other CAR-T constructs in GBM show similar results. T-cells with a CAR against HER2 led to treatment-related grade 2 neurotoxicity in 2 of 17 patients, characterized by seizure (in both) and headache (in 1).^86^ Intracranial delivery of CAR-T cells targeting interleukin-13 receptor alpha 2 (IL13Rα2) in 3 patients was associated with grade 3 headache in one patient and a grade 3 neurologic event characterized by shuffling gait and tongue deviation in a second patient.^87^ Based on published data it is not clear that these patients met criteria for ICANS, though neurologic events could be related to localized T-cell activation and intracranial cytokine release. These cases highlight the difficulty of neurologic adverse event adjudication in patients with aggressive brain tumors. Further study in larger cohorts is needed to characterize the scope of neurotoxicity in patients with GBM who receive CAR-T cell therapy.

## Discussion

Prompt recognition of ICI and CAR-T cell-related neurotoxicity is critical to prevent associated morbidity and mortality. This requires an understanding of the toxicity syndromes that occur, which have unique features that differ from idiopathic disease. We have reviewed these syndromes, discussed specific challenges patients with brain metastases, and presented a framework for diagnosis that includes consideration of the therapy regimen, timing of symptom onset, presence of non-neurologic irAEs, pre-existing neurologic disease, and symptom specific features.

Since the first ICI was approved in 2011, the landscape of cancer treatment has changed dramatically. There are currently 8 ICIs and 5 CAR-T cell therapies approved, with growing lists of indications that continue to expand. In addition, the pipeline holds next generation inhibitors targeting new inhibitory checkpoints and many potential adoptive cell therapies. The next ten years promise continued advancement, with new classes of emerging immunotherapies, and the goal of addressing the lack of tumor response or the development of resistance to current agents. The first oncolytic virus talimogene laherparepvec (T-VEC) was approved for melanoma in 2015, and other viruses are in development for lung, prostate, cervical, and other solid cancers.^88^ The therapeutic vaccine sipuleucel-T is currently approved for prostate cancer, with active development of vaccines for ovarian cancer, renal cell carcinoma, and melanoma.^88^ Work to develop personalized vaccines, including leveraging mRNA technologies, is also underway.^88^ Oncolytic viruses and therapeutic vaccines are being studied in combination with targeted antibodies and ICIs.^88^ Going forward, we can expect to see growing use of immunotherapies in combination, which may have implications for autoimmune toxicities.

With more widespread use of existing immunotherapies and development of novel ways to harness the immune system for anti-tumor activity, the number of patients affected by neurotoxicity will continue to grow. The challenges in diagnosing these toxicities in patients with brain metastases highlight the need for biomarkers, clear disease definitions, and improved diagnostics. There is an urgent need to understand pathogenic mechanisms, predictors, and optimal treatments of these toxicities, so that we can manage them without sacrificing anti-tumor efficacy.
